# A Comprehensive Analysis of miRNA/isomiR Expression with Gender Difference

**DOI:** 10.1371/journal.pone.0154955

**Published:** 2016-05-11

**Authors:** Li Guo, Tingming Liang, Jiafeng Yu, Quan Zou

**Affiliations:** 1 Department of Bioinformatics, School of Geographic and Biologic Information, Nanjing University of Posts and Telecommunications, Nanjing, China; 2 Jiangsu Key Laboratory for Molecular and Medical Biotechnology, College of Life Science, Nanjing Normal University, Nanjing, China; 3 Shandong Provincial Key Laboratory of Functional Macromolecular Biophysics, Institute of Biophysics, Dezhou University, Dezhou, China; 4 School of Computer Science and Technology, Tianjin University, Tianjin, China; 5 State Key Laboratory of Medicinal Chemical Biology, NanKai University, Tianjin, China; Huazhong University of Science and Technology, CHINA

## Abstract

Although microRNAs (miRNAs) have been widely studied as epigenetic regulation molecules, fewer studies focus on the gender difference at the miRNA and isomiR expression levels. In this study, we aim to understand the potential relationships between gender difference and miRNA/isomiR expression through a comprehensive analysis of small RNA-sequencing datasets based on different human diseases and tissues. Based on specific samples from males and females, we determined that some miRNAs may be diversely expressed between different tissues and genders. Thus, these miRNAs may exhibit inconsistent and even opposite expression between males and females. According to deregulated miRNA expression profiles, some dominantly expressed miRNA loci were selected to analyze isomiR expression patterns using rates of dominant isomiRs. In some miRNA loci, isomiRs showed statistical significance between tumor and normal samples and between males and females samples, suggesting that isomiR expression patterns are not always invariable but may vary between males and females, as well as among different tissues, tumors, and normal samples. The divergence implicates the fluctuation in the expression of miRNA and its detailed expression at the isomiR levels. The divergence also indicates that gender difference may be an important factor that affects the screening of disease-associated miRNAs and isomiRs. This study suggests that miRNA/isomiR expression and gender difference may be more complex than previously assumed and should be further studied according to specific samples from males or females.

## Introduction

A class of small non-coding RNAs (ncRNAs), microRNAs (miRNAs), has been widely studied as endogenous negative regulatory molecules to control post-transcriptional gene expression [1]. miRNAs play a pivotal role in coding-non-coding RNA regulatory networks, and many abnormally expressed miRNAs were proven to be associated with human diseases, particularly in promoting carcinogenesis in some cancers [2, 3]. Although a single miRNA sequence is believed to come from a miRNA locus, a series of heterogeneous sequences have been widely detected and identified in both human and plant miRNAs by comprehensively analyzing high-throughput sequencing datasets [4–9]. Some isomiRs have been proven as functional small RNAs by associating with target mRNAs [6, 10–13] and influencing miRNA stability [14] or effectiveness [15, 16]. More studies focus on these multiple miRNA variants because of the versatile roles of isomiRs, particularly those dominantly expressed isomiRs, and isomiRs with 5’ variation and/or 3’ additions. Simultaneously, the analysis of miRNA-miRNA or isomiR-isomiR interaction combined with homologous and/or clustered miRNAs is necessary from the miRNA/isomiR levels [17], and miRNA may be thoroughly studied from the detailed isomiR expression.

Some relevant biological molecules, including proteomes, mRNA, and miRNA, were reported to be differently expressed or enriched between samples from males and females [18–21]. Specifically, some miRNAs were determined to have various expressions between genders [22, 23], and the promoter methylation of miR-137 was validated as a female-associated molecule [24]. Loher et al. [25] found that expressions of many isomiRs diverged between males and females. These findings demonstrate that miRNA or isomiR expression may be related with gender difference, and gender-associated miRNA or isomiR expression and function should be not ignored. Typical analyses and studies always disregard the relationship of miRNA or isomiR expression and gender difference, and some gender-related miRNAs may be ignored or balanced using the typical analysis. However, fewer systematic studies are carried out, particularly those that are based on the miRNA or isomiR biogenesis and the expression between males and females across different diseases and tissues.

In this study, based on the potential importance and relationships of miRNA/isomiR expression and gender difference, a comprehensive analysis was performed using specific and common diseases in males and females. The study aimed to explore the divergence of miRNA/isomiR expression profiles and gender difference at the miRNA and isomiR levels, respectively. Specifically, the potential expression divergence was analyzed between different tissues, tumor and normal groups, and isomiR expression patterns in different tissues and genders. According to miRNA/isomiR characteristics and gender difference, the study may provide more information and implications for studies on miRNA and isomiR, particularly specific miRNA and isomiR expression profiles in specific human diseases.

## Materials and Methods

### Flowchart

**Fig 1:**
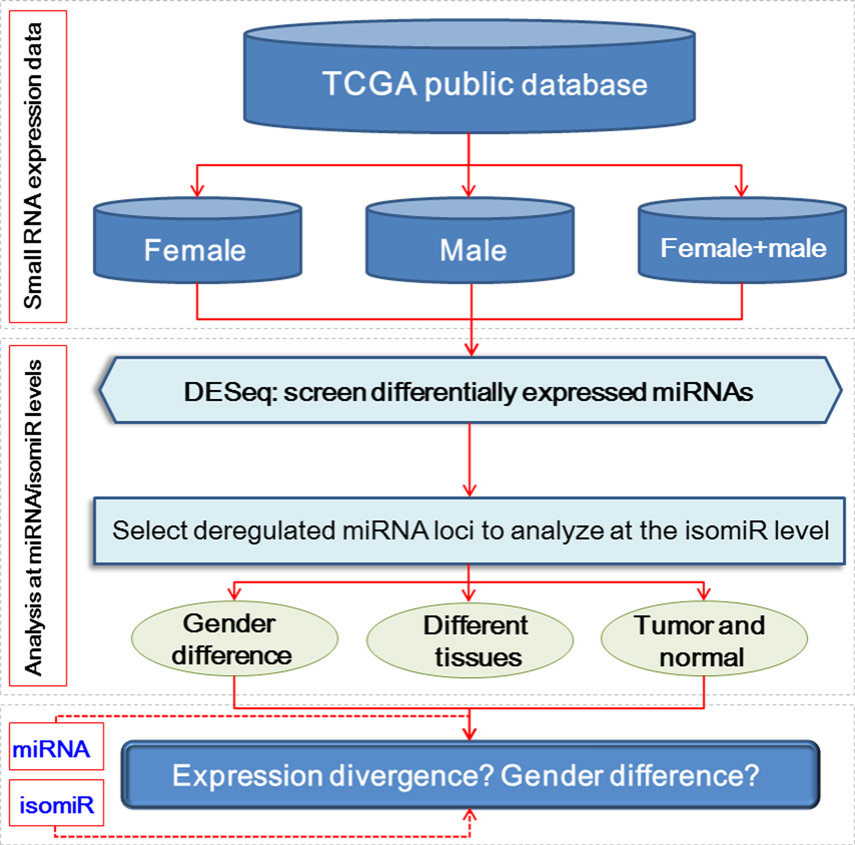
The flowchart to analyze miRNA/isomiR in the study.

A flowchart of a comprehensive analysis of miRNA and isomiR expression was indicated in Fig 1, which would contribute in determining the relationship of miRNA/isomiR expression and gender difference.

### Source data

The small RNA deep sequencing datasets used in this work were obtained from The Cancer Genome Atlas (TCGA) pilot project (https://tcga-data.nci.nih.gov/tcga/), and all the data were sequenced by the Illumina HiSeq sequencing platform. These datasets included small RNA sequencing of tumor and control samples from females (a specific disease in females, uterine corpus endometrial carcinoma (UCEC)), males (specific disease in males, prostate adenocarcinoma (PRAD)), and females and males (common diseases in males and females, lung squamous cell carcinoma (LUSC) and thyroid carcinoma (THCA)). Equal sample sizes were randomly obtained from the corresponding tumor samples based on the same and similar characteristics because of the divergence of sample sizes between tumor and normal samples (S1 Table).

### Sequence and expression analysis

Small RNA expression profiles were first collected from TCGA (the original sequencing data were analyzed by mapping analysis), and these profiles were further analyzed at the miRNA and isomiR levels. Differentially expressed miRNAs were obtained using DESeq package [26] according to miRNA expression profiles if the profiles were statistically diverged and had relative abundant enrichment levels. Based on the obtained abnormal miRNA expression profiles, some abundantly expressed miRNA loci were selected to further screen the isomiR expression profiles (Fig 1).

According to all isomiRs from a specific miRNA locus, the relative expression level of each isomiR type was estimated using the percentage in each individual. The average percentages and standard deviations were used to describe isomiR expression according to the multiple individuals in a sample. The rates of the top four dominant isomiRs were selected to estimate the isomiR expression patterns based on several dominant isomiRs in the miRNA locus [4,9]. Specifically, the rates of dominant isomiRs were employed, including the rate of the most dominant and secondary dominant isomiRs, the rate of the most dominant and the third dominant isomiRs, and the rate of the most dominant and the fourth dominant isomiRs.

### Statistical analysis

Venny’s distribution of deregulated expressed miRNAs was constructed using Venny web server 2.0 (http://bioinfogp.cnb.csic.es/tools/venny/index.html). The expression data at the miRNA/isomiR levels were described as mean ± standard deviation ($\bar{x}$ ± SD). Differentially expressed miRNA species were collected by utilizing a DESeq package that contained statistical analysis, including multiple comparison correction using false discovery rate (FDR). The rates of dominant isomiRs were adopted to estimate isomiR expression, and the distribution of each rate followed the normal distribution. A *t* test was used to estimate the difference between tumor and relevant normal tissues, and between samples from males and females in each disease. Furthermore, ANOVA analysis was employed to estimate the isomiR expression difference among different groups. If the *P* or *P*_adj_ values (the associated FDR) were less than 0.05, the difference would be considered a statistical difference.

## Results

### Expression analysis at the miRNA level

**Fig 2:**
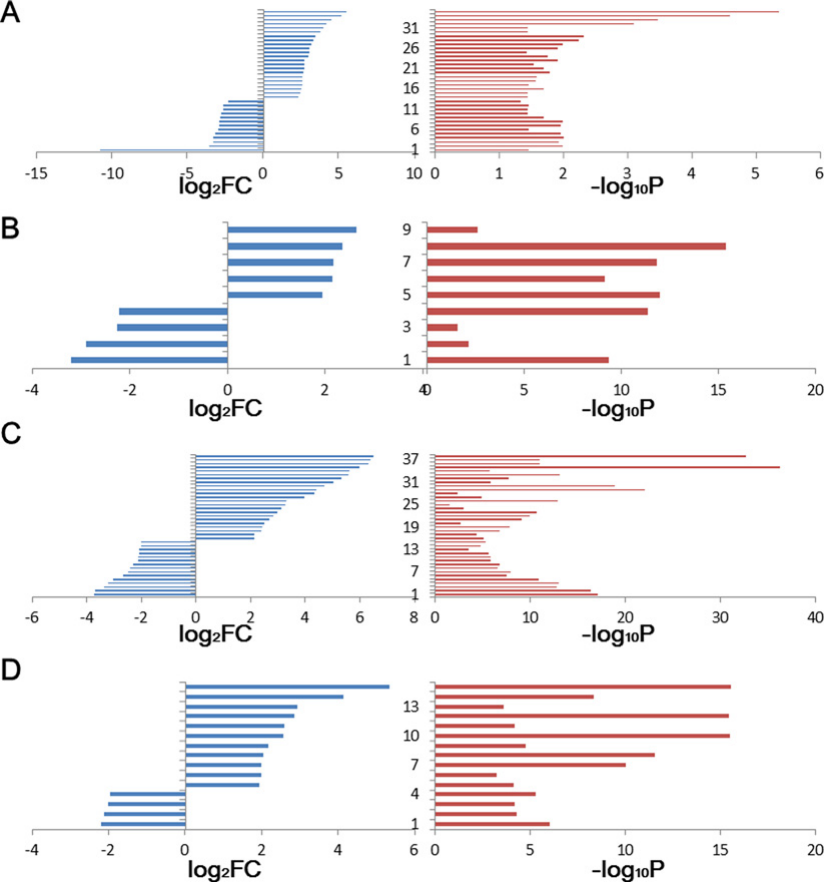
Distributions of deregulated miRNAs in the 4 kinds of diseases. A: UCEC; B: PRAD; C: LUSC; D: THCA.

Based on relative expression levels at the miRNA level (the average sequence counts were more than 50 in tumor or normal tissues) (Fig 1 and S1 Table), a series of miRNAs were collected as significant abnormally expressed species in tumor tissues (|Log_2_(Fold-change)| ≥ 2.0 and *P*_adj_ < 0.05), and the up-regulated miRNAs in these tumor samples were dominant (Fig 2). A total of 38 deregulated miRNAs were obtained in PRAD and followed by 35 miRNAs in UCEC, whereas less deregulated expressed miRNAs were detected in PRAD (9) and LUSC (15) according to the standard (S1A Fig). Among these deregulated miRNAs, less common species were detected across different tissues (S1B Fig).

Based on deregulated miRNAs and their relative expression levels (average sequence counts were more than 5000 in tumor or normal tissues, which could ensure further analysis at the isomiR levels), some typical miRNA loci were presented in S2 Table. Further analysis was performed according to the female and male samples in the same tissues (Table 1 and S2 Fig). A series of miRNAs were identified as deregulated species between different genders with significant difference. However, specific deregulated species were quite dominant (S2 Fig). Some specific miRNA, such as miR-375 in LUSC, exhibited opposite expression patterns in females (up-regulated, log_2_FC = 1.53) and males (down-regulated, log_2_FC = -2.73), whereas the miRNA was identified as normally expressed miRNA based on the mixed samples (log_2_FC = -0.35). Interestingly, the specific miRNA, miR-375, was identified as up-regulated miRNA in female-specific UCEC (log_2_FC = 2.61, *P* = 0.0262), whereas it was also determined as up-regulated miRNA without statistical significance in male-specific PRAD (log_2_FC = 2.15, *P* = 6.93E-10) (S2 Table).

**Table 1:** Screened deregulated miRNAs in males (n = 12) and females groups (n = 12).

	Down-regulated			Up-regulated		
Disease sample	miRNA	Log_2_(FC)	*P*_*adj*_	miRNA	Log_2_(FC)	*P*_*adj*_
**Female-LUSC**	miR-144	-5.05	0.0012	miR-205	7.31	2.59E-06
	miR-451	-5.06	0.0012	miR-1269	8.94	3.92E-06
	miR-486	-4.75	0.0025	miR-210	5.18	0.0005
	miR-30a	-3.09	0.0427	miR-183	3.74	0.0230
	miR-338	-3.16	0.0433	miR-9	6.79	0.0300
**Male-LUSC**	miR-451	-4.19	6.81E-07	miR-205	7.68	2.59E-22
	miR-30a	-3.23	4.48E-05	miR-210	5.15	1.45E-05
	miR-101	-3.11	0.0002	miR-183	3.27	9.68E-05
	miR-30d	-3.03	0.0009	miR-9	5.62	0.0026
	miR-375	-2.73	0.0023	miR-203	2.24	0.0046
	miR-140	-2.63	0.0047	miR-182	2.38	0.0078
	miR-29c	-2.58	0.0060	miR-141	2.05	0.0324
	miR-100	-2.25	0.0107			
	miR-143	-2.09	0.0175			
	miR-181a	-2.22	0.0465			
**Female-THCA**	miR-451	-2.51	0.0002	miR-183	2.67	9.42E-05
	miR-486	-2.40	0.0002	miR-221	2.70	0.0006
	miR-144	-2.26	0.0005	miR-182	2.35	0.0012
**Male-THCA**				miR-146b	5.97	4.69E-05
				miR-221	3.29	0.0279

*Note*: The sample size is selected according to the actual distribution of females and males in the normal sample, and the relevant selected individuals are collected according to the principle of homogeneous.

### Expression analysis at the isomiR level in different diseases

**Fig 3:**
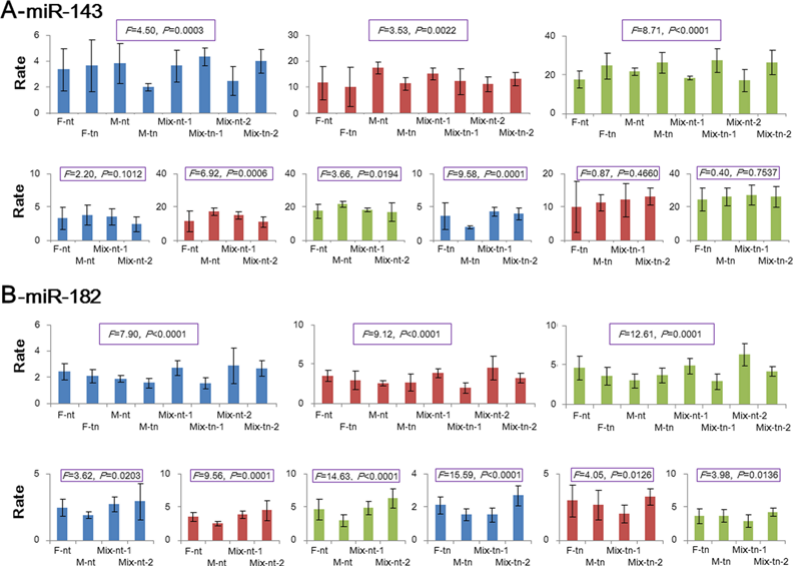
Expression distribution of rates of dominant isomiRs across different groups. The *F* statistic and *P* values are also presented using ANOVA analysis among different groups. F-nt: female-UCEC-nt; F-tn: female-UCEC-tn; M-nt: male-PRAD-nt; M-tn: male-PRAD-tn; Mix-nt-1: mixed-LUSC-nt; Mix-tn-1: mixed-LUSC-tn; Mix-nt-2: mixed-THCA-nt; Mix-tn-2: mixed-THCA-tn. The blue bar indicates the rate of the most dominant isomiR and the secondary dominant isomiR, the red bar indicates the rate of the most dominant isomiR and the third dominant isomiR, and the green bar indicated the rate of the most dominant isomiR and the fourth dominant isomiR.

Deregulated miRNAs were analyzed based on the sum of all varied sequences, which were also called isomiR sequences in the corresponding miRNA gene loci, whereas the detailed sequences were not further analyzed. According to versatile biological roles and potential function, these isomiR sequences should be further involved in the in-depth analysis. Therefore, some common deregulated miRNA loci were selected to perform isomiR expression analysis based on gender-specific diseases and the common diseases, including down-regulated miR-143 and up-regulated miR-182 (S2 Table). The rates of dominant isomiRs from the two miRNA loci exhibited significant difference across the eight groups (Fig 3). Based on all the normal and tumor groups across different tissues, the rates of dominant isomiRs showed diverse expression except for miR-143 in normal groups. The rate of the most dominant and secondary dominant isomiRs showed a statistical difference among normal and tumor groups (Fig 3).

For each pair of tumor, the corresponding normal samples, and male and female samples, a relevant *t* test was used to estimate the difference. As expected, isomiR expression may show a significant difference between tumor and control samples, particularly for miR-143 in male-specific PRAD disease and miR-182 in LUSC disease (S3 Table). The two miRNA loci showed diverse divergence between other pairwise samples, and they may also exhibit significant difference between various tissues, although the difference was more common between males and females. miRNA locus may generate inconsistent isomiR expression patterns between different gender-specific diseases, between different common diseases, and between males and females (S3 Table).

### Expression analysis at the isomiR level between males and females

We further selected the common dominantly expressed miRNA loci to track isomiR expression patterns between different genders based on the same human diseases between males and females, including the diseases LUSC and THCA. Some miRNAs were collected to understand the isomiR expression in LUSC, including down-regulated miRNAs (miR-451 and miR-30a) and up-regulated miRNAs (miR-205, miR-210, miR-183, and miR-9). In THCA, only one up-regulated miR-221 was collected, and the further extension of down-regulated miR-451 was selected although it was identified as down-regulated miRNA without statistical significance in the male–THCA group (log_2_FC = -1.83, *P*_*adj*_ = 0.6557).

**Fig 4:**
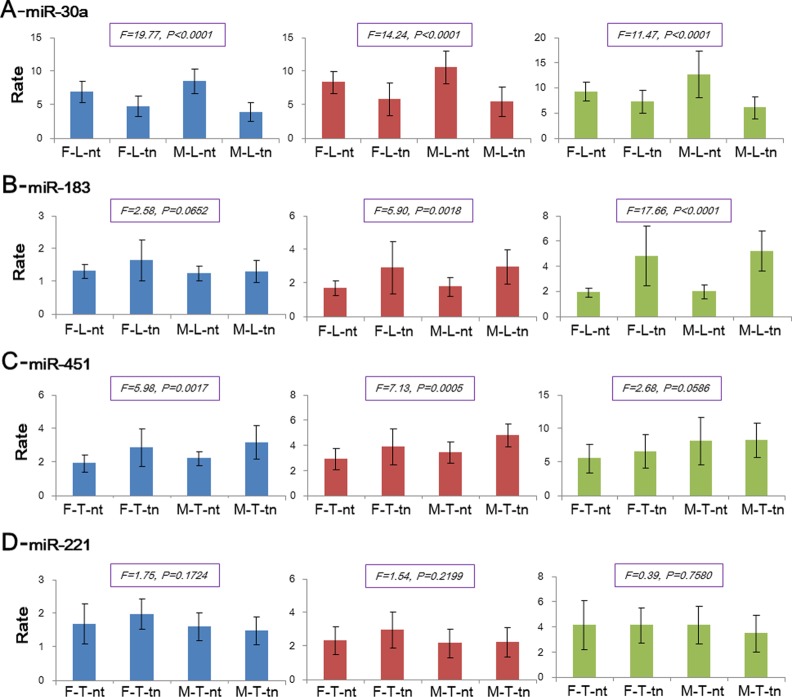
Expression distribution of dominant isomiRs across female and male groups in the specific common diseases. F-L-nt: female LUSC control group; F-L-tn: female LUSC tumor group; M-L-nt: male LUSC control group; M-L-tn: male LUSC tumor group; F-T-nt: female THCA control group; F-T-tn: female THCA tumor group; M-T-nt: male THCA normal group; M-T-tn: male THCA tumor group. Other detailed annotations can be found in Fig 3.

Expression analysis showed that most of these deregulated miRNAs had similar isomiR expression patterns across the four groups with pairwise male and female groups. However, miR-30a loci exhibited statistical difference in all the most dominant isomiRs (*P* < 0.0001), and other miRNAs may show significant difference in some certain rates of dominant isomiR species (Fig 4). For example, in the miR-183 locus, the rate of the most dominant and secondary dominant isomiRs was similar across different groups, but other rates showed significant difference (*P* = 0.0018 and *P* < 0.0001, respectively, Fig 4). In the miR-451 locus, except for the rate of most dominant and fourth dominant isomiRs, other rates differed, which also demonstrated that the miRNA locus could generate different isomiR expression profiles. Further statistical analysis based on pairwise male and female samples and tumor and normal samples in specific disease were performed using an unpaired *t* test. The isomiR expression of the miR-30a locus showed statistical difference. The rate of the most dominant and secondary dominant isomiRs in miR-451 differed between normal and tumor samples in males and females, whereas other miRNA loci exhibited similar isomiR expression between normal and tumor samples (Table 2). For the expression between males and females, miR-30a showed significant expression divergence in normal tissues, whereas no significant difference was detected in tumor samples. The rate of the most dominant and secondary dominant isomiRs varied between tumors from males and females (*t* = 2.4642, *P* = 0.0254), whereas other miRNA loci showed similar isomiR expression (Table 2).

**Table 2:** Statistical analysis between paired tumor and normal samples or male and female samples using *t* test in Fig 4.

miRNA	Tissues	The first/second	The first/third	The first/fourth
**miR-30a**	**Female:** LUSC-nt & LUSC-tn	*t* = 3.3228, *P* = 0.0031	*t* = 2.9598, *P* = 0.0072	*t* = 2.2636, *P* = 0.0338
	**Male:** LUSC-nt & LUSC-tn	*t* = 6.7896, *P*<0.0001	*t* = 5.4469, *P*<0.0001	*t* = 4.4999, *P* = 0.0002
	**LUSC-nt:** Female & male	*t* = -2.2102, *P* = 0.0378	*t* = -2.6317, *P* = 0.0152	*t* = -2.3787, *P* = 0.0265
	**LUSC-tn:** Female & male	*t* = 1.4378, *P* = 0.1646	*t* = 0.3988, *P* = 0.6939	*t* = 1.3850, *P* = 0.1799
**miR-183**	**Female:** LUSC-nt & LUSC-tn	*t* = -1.6891, *P* = 0.1053	*t* = -2.6513, *P* = 0.0146	*t* = -4.1722, *P* = 0.0004
	**Male:** LUSC-nt & LUSC-tn	*t* = -0.4891, *P* = 0.6296	*t* = -3.4908, *P* = 0.0021	*t* = -6.7018, *P* = 0.0001
	**LUSC-nt:** Female & male	*t* = 0.8350, *P* = 0.4127	*t* = -0.3776, *P* = 0.7093	*t* = -0.2330, *P* = 0.8179
	**LUSC-tn:** Female & male	*t* = 1.6558, *P* = 0.1119	*t* = -0.0634, *P* = 0.9500	*t* = -0.4613, *P* = 0.6491
**miR-451**	**Female:** THCA-nt & THCA-tn	*t* = -2.6401, *P* = 0.0153	*t* = -2.0416, *P* = 0.0540	*t* = -1.0727, *P* = 0.2956
	**Male:** THCA-nt & THCA-tn	*t* = -3.0656, *P* = 0.0057	*t* = -3.7824, *P* = 0.0010	*t* = -0.0637, *P* = 0.9498
	**THCA-nt:** Female & male	*t* = -1.4825, *P* = 0.1524	*t* = -1.4711, *P* = 0.1554	*t* = -2.1874, *P* = 0.0396
	**THCA-tn:** Female & male	*t* = -0.7010, *P* = 0.4910	*t* = -1.7565, *P* = 0.0936	*t* = -1.5505, *P* = 0.1360
**miR-221**	**Female:** THCA-nt & THCA-tn	*t* = -1.2174, *P* = 0.2384	*t* = -1.4958, *P* = 0.1511	*t* = 0.0682, *P* = 0.9463
	**Male** THCA-nt & THCA-tn	*t* = 0.7464, *P* = 0.4646	*t* = -0.1590, *P* = 0.8753	*t* = 1.0025, *P* = 0.3287
	**THCA-nt:** Female & male	*t* = 0.3472, *P* = 0.7317	*t* = 0.5005, *P* = 0.6217	*t* = 0.0353, *P* = 0.9722
	**THCA-tn:** Female & male	*t* = 2.4642, *P* = 0.0254	*t* = 1.5584, *P* = 0.1387	*t* = 0.9358, *P* = 0.3633

Other deregulated miRNAs were only analyzed at the isomiR levels in tumor or normal tissues of males and females because these miRNAs were rarely detected in the corresponding normal or tumor tissues. All of these miRNAs showed similar expression using the three kinds of rates, although some of them tended to involve in a larger tendency of dispersion (S3 Fig), particularly for the rate of the most dominant and the fourth dominant isomiRs.

The results shown here are in whole based upon data generated by the TCGA Research Network: http://cancergenome.nih.gov/.

## Discussion

The crucial roles of miRNAs in various diseases have attracted many researches, and disease-related miRNAs, including their target mRNAs, can be predicted based on biological interaction network [27–35]. Systematic analysis and study of roles and interactions of miRNAs in specific biological process, such as cell death [36–38], cell proliferation [39, 40], have been performed in diverse human diseases. The phenomenon of multiple isomiRs further enriches the miRNA/isomiR study, and disease-related isomiRs may be next crucial markers to study occurrence and development of diseases. Although the phenomenon of isomiRs was first detected by analyzing deep-sequencing small RNA datasets, these miRNA variants with various sequences and expressions gained the attention of researchers based on their versatile biological functions [6, 10–16]. In the specific miRNA locus, different isomiRs may demonstrate diverse deregulated expression patterns and adverse expression, although these sequence-related isomiRs may have close functional relationships, including co-regulating target mRNAs. The typical analysis of miRNAs without considering the isomiR level may be a partial solution, and the comprehensive analysis from the isomiR level is quite important, particularly because some deregulated miRNAs are prone to detect abnormal isomiR expression patterns [41]. Loher et al. reported that isomiR expression profiles may be population-dependent and gender-dependent [25], particular for the diversion of gene expression and protein between males and females [18–21]. For small non-coding RNAs, more studies focused on the gender-specific miRNAs or miRNA expression with gender difference [42–45], but systematic analysis of isomiR expression based on miRNA loci and studies based on deregulated miRNA loci are rare. Is the gender-dependent isomiR expression profile highly significant? This study may provide more implications for miRNA and isomiR studies in gender-relevant samples. Based on these results and our previous studies, we aimed to explore the potential isomiR expression in different human diseases and genders, including male-specific and female-specific diseases and diseases for both genders. Further analysis was performed between males and females using the common tissues and diseases.

Diversely deregulated miRNA and isomiR expression profiles can be found in specific diseases from males and females (Fig 2, S2 Table, and S1 Fig). Although some miRNAs are identified as common deregulated species in gender-specific diseases, such as deregulated miR-182 and miR-183, these miRNAs always exhibit different levels of up- or down-regulated expression (S2 Table). Most of these miRNAs were studied as crucial miRNAs in the occurrence and development of some diseases. For example, miR-182 and miR-183 are identified as oncogenic miRNAs and contribute to early breast cancer development [46]. Specific miRNAs may show opposite expression patterns between females and males, whereas they may be identified as normally expressed miRNAs using the mixed samples. Thus, some abnormally expressed miRNAs may be disregarded or balanced in typical analysis using mixed genders where gender may be an important factor in examining miRNA. Disease-associated miRNAs are always selected and identified from these deregulated miRNAs, and inconsistent and abnormal expression profiles reveal that the small RNA expression may be influenced by gender difference. Diverse miRNA expression profiles lead to diverse isomiR profiles, which complicate and interrupt further experimental validation of disease-associated miRNAs.

IsomiR expressions in selected common deregulated miRNA loci, including miR-143 and miR-182, differ across the four groups with different diseases (*P* < 0.01, Fig 3). Analyses of isomiRs in normal and tumor samples showed that rates of dominant isomiRs may be similar or diverged among different genders and tissues (from different diseases), thus suggesting that isomiR expressions may diverge between genders and tissues (Fig 3). The analysis also focuses gender difference and tissue difference, which may contribute to various isomiR expressions. After further pairwise comparisons of isomiRs between normal and tumor samples, two miRNAs may be significantly diverged between some tumor and normal samples (*P* < 0.05) or have similar expression between tumor and normal samples (*P* > 0.05, S3 Table). The three rates of dominant isomiRs also have an inconsistent expression. However, the rate of the top-dominant isomiRs may be most important mark to estimate the isomiR expression (the top two dominant isomiRs may possess nearly 80% expression in certain miRNA loci). These findings indicate that the selected deregulated miRNA loci may generate inconsistent isomiR expression between genders, normal and tumor samples, and different tissues. Although analysis at the miRNA level indicates that these miRNAs are abnormally expressed in tumor samples, the detailed and real expression at the isomiR levels may vary. The divergence of isomiR expression patterns implies that isomiRs may be diverged between different tissues and genders, which may affect the selection of deregulated miRNAs for further experimental validation. More importantly, in these multiple isomiRs, some isomiRs with varied or shifted “seed sequences” may also have higher enrichment levels. However, the changed functional regions may result in new target mRNAs or binding sites with target mRNAs. These isomiRs may enrich the function in the miRNA locus, and analysis at the isomiR levels may be particularly considered in studies on miRNAs.

To further understand the potential expression divergence between males and females in the same tissues, two common diseases in males and females (LUSC and THCA) were selected to analyze isomiRs in some miRNA loci between males and females. These selected miRNAs were identified as deregulated species. However, not all of these miRNA loci showed diverged isomiR expression across different groups (such as miR-183 and miR-221, Fig 4). Similarly, pairwise comparisons of isomiRs demonstrate that miR-30a and miR-221 diverged between genders; and miR-30a and miR-451 are diverged between normal and tumor samples (Table 2). These findings suggest that isomiRs from a specific miRNA locus may vary between males and females and between normal and tumor samples in the specific samples. However, typical analysis at the miRNA level could disregard this divergence, particularly when mixed samples from females and males are adopted. The final screening of deregulated miRNAs would not reflect the real expression and change at the isomiR levels. Analysis at the isomiR level can lead to inconsistent expression patterns although these isomiRs are generated from the specific miRNA locus. These multiple isomiRs always have close functional relationships (the same seed sequences or shifted seed sequence) [41]. A comprehensive analysis at the miRNA and isomiR levels is necessary to further reveal the complex small RNAs.

Taken together, this study determined that miRNA and isomiR expressions may diverge between normal and tumor samples and between females and males. These expression divergences suggest that gender may be an important factor in miRNA and isomiR expression. A canonical analysis of miRNA/isomiR without considering gender difference may disregard some deregulated miRNAs, and isomiRs in miRNA loci, which would increase the false positive rate. Candidate disease-associated miRNAs should be screened and examined better based on gender-difference at the isomiR levels.

## Supporting Information

S1 Fig The distribution of deregulated expressed miRNA species in the different tumor tissues. (TIF)

S2 Fig The distribution of deregulated expressed miRNA species in different genders. (TIF)

S3 Fig Expression distribution of dominant isomiRs across female and male groups in LUSC. These deregulated miRNA loci are only abundantly expressed in tumor or control groups. The detailed annotations can be found in Figs 3 and 4.
(TIF)

S1 Table Selected small RNA sequencing datasets from the TCGA database. (DOCX)

S2 Table Selected deregulated miRNA loci in the 4 groups. (DOCX)

S3 Table Statistical analysis results between paired tumor and normal samples, and male and females samples, using the *t* test. (DOCX)
