# Supplementary figures and images for: A Comprehensive Analysis of miRNA/isomiR Expression with Gender Difference
Source: PLoS One. 2016 May 11;11(5):e0154955. doi: 10.1371/journal.pone.0154955 (PMC4864079; doi:10.1371/journal.pone.0154955)

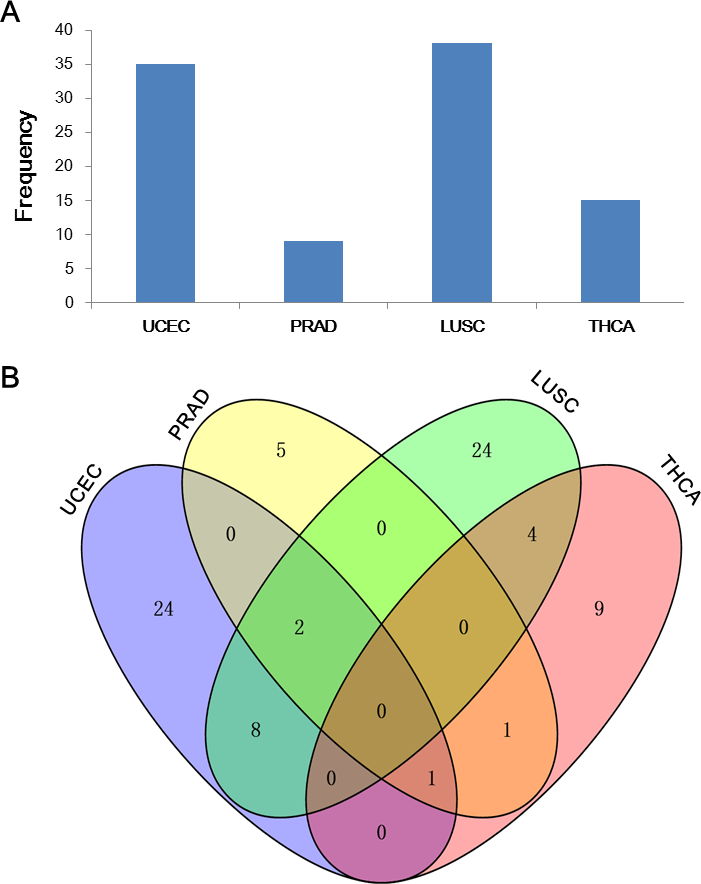

Supplement: S1 Fig — (TIF) [file pone.0154955.s001.tif]

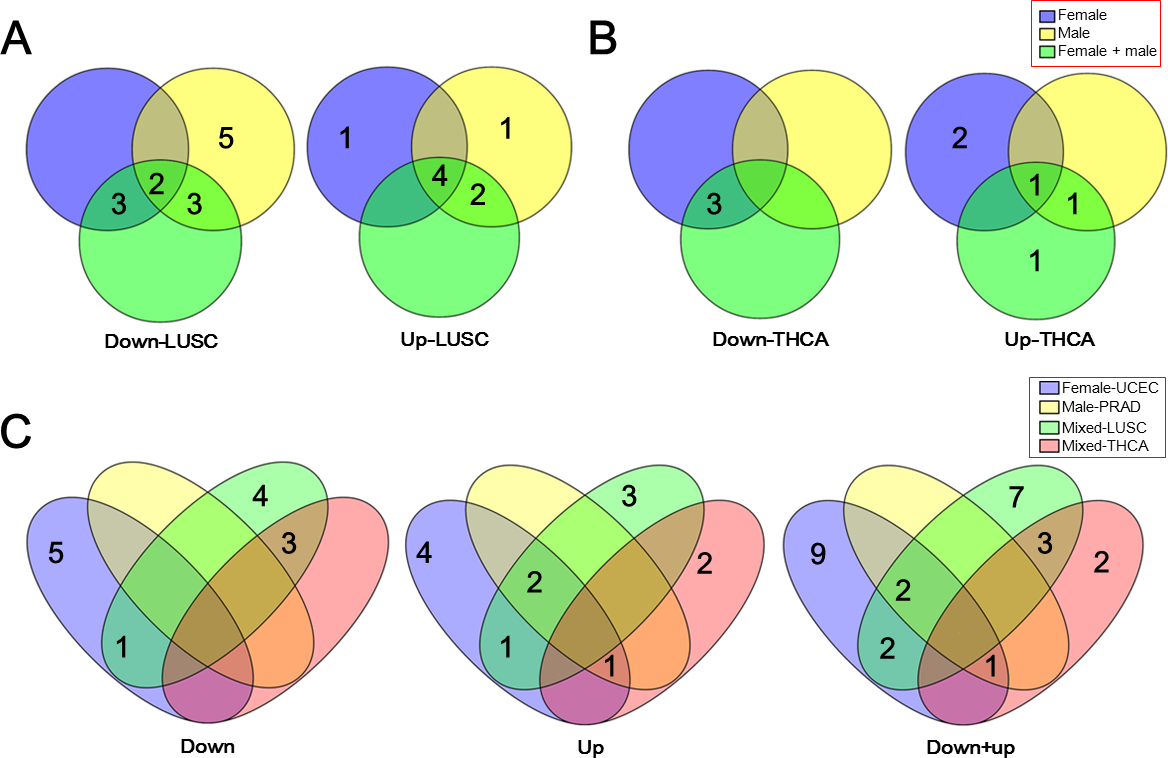

Supplement: S2 Fig — (TIF) [file pone.0154955.s002.tif]

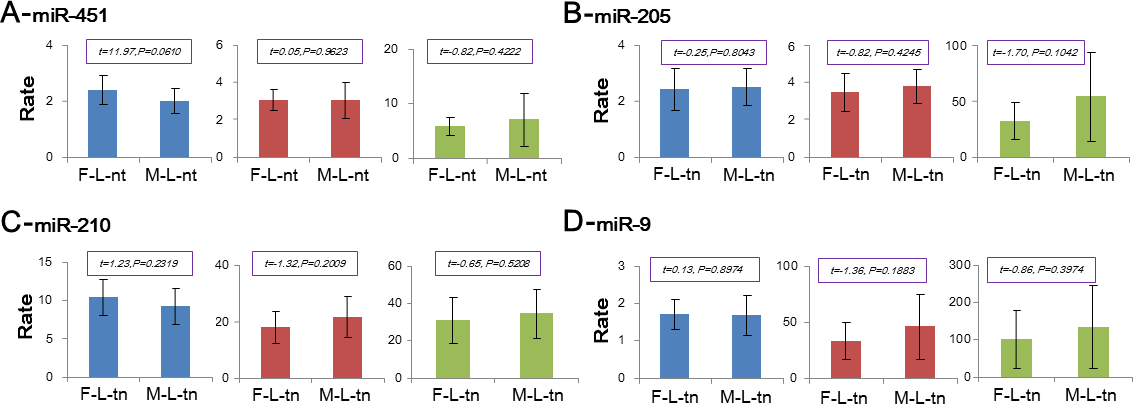

Supplement: S3 Fig — These deregulated miRNA loci are only abundantly expressed in tumor or control groups. The detailed annotations can be found in Figs 3 and 4. (TIF) [file pone.0154955.s003.tif]
