# Supplementary material for: A Comprehensive Analysis of miRNA/isomiR Expression with Gender Difference
Source: PLoS One. 2016 May 11;11(5):e0154955. doi: 10.1371/journal.pone.0154955 (PMC4864079; doi:10.1371/journal.pone.0154955)
Supplement: S1 Table — (DOCX) [file pone.0154955.s004.docx]

**Table S1. Selected small RNA sequencing datasets from the TCGA database.**

| **Origin** | **Disease** | **TN** | **NT** |
| --- | --- | --- | --- |
| Female | UCEC | 21 | 21 |
| Male | PRAD | 52 | 52 |
| Male and female | LUSC | 45 | 45 |
| Male and female | THCA | 59 | 59 |
| Total |  | 177 | 177 |

*Note:* TN: tumor, matched normal; NT: normal, matched tumor; UCEC: uterine corpus endometrial carcinoma, is a kind of female-specific disease; PRAD: Prostate adenocarcinoma, is a kind of male-specific disease; LUSC: Lung squamous cell carcinoma; THCA: Thyroid carcinoma.
