# Supplementary material for: A Comprehensive Analysis of miRNA/isomiR Expression with Gender Difference
Source: PLoS One. 2016 May 11;11(5):e0154955. doi: 10.1371/journal.pone.0154955 (PMC4864079; doi:10.1371/journal.pone.0154955)
Supplement: S2 Table — (DOCX) [file pone.0154955.s005.docx]

**Table S2. Selected deregulated miRNA loci in the four groups.**

| **Disease sample** | **Down-regulated** | | | **Up-regulated** | | |
| --- | --- | --- | --- | --- | --- | --- |
|  | **miRNA** | **Log_2_(FC)** | ***P_adj_*** | **miRNA** | **Log_2_(FC)** | ***P_adj_*** |
| **Female-specific-UCEC** | miR-143 | -2.85 | 0.0202 | miR-182 | 4.23 | 0.0008 |
|  | miR-100 | -3.30 | 0.0118 | miR-183 | 5.17 | 2.47E-05 |
|  | miR-145 | -3.14 | 0.0111 | miR-203 | 3.00 | 0.0171 |
|  | let-7c | -2.62 | 0.0357 | miR-375 | 2.61 | 0.0262 |
|  | miR-99a | -2.74 | 0.0357 | miR-1307 | 2.71 | 0.0202 |
|  | miR-424 | -2.88 | 0.0103 | miR-200a | 3.20 | 0.0100 |
|  |  |  |  | miR-200b | 2.59 | 0.0345 |
|  |  |  |  | miR-210 | 3.32 | 0.0057 |
| **Male-specific-PRAD** |  |  |  | miR-182 | 2.37 | 4.00E-16 |
|  |  |  |  | miR-183 | 2.18 | 1.42E-12 |
|  |  |  |  | miR-375 | 2.15 | 6.93E-10 |
| **Mixed-LUSC** | miR-143 | -2.08 | 2.49E-06 | miR-182 | 2.40 | 1.48E-07 |
|  | miR-30a | -3.01 | 1.12E-11 | miR-183 | 2.71 | 8.04E-10 |
|  | miR-101 | -2.49 | 1.17E-08 | miR-141 | 2.14 | 7.79E-06 |
|  | miR-30d | -2.31 | 1.75E-07 | miR-205 | 5.99 | 4.91E-37 |
|  | miR-451 | -3.21 | 1.06E-13 | miR-9 | 5.05 | 1.52E-06 |
|  | miR-338 | -2.12 | 1.45E-06 | miR-210 | 4.42 | 8.67E-23 |
|  | miR-486 | -3.70 | 4.06E-17 |  |  |  |
|  | miR-144 | -3.72 | 8.10E-18 |  |  |  |
| **Mixed-THCA** | miR-144 | -1.99 | 6.15E-05 | miR-221 | 2.86 | 3.34E-16 |
|  | miR-486 | -2.11 | 5.03E-05 | miR-146b | 5.35 | 2.69E-16 |
|  | miR-451 | -2.18 | 9.28E-07 | miR-375 | 2.61 | 6.69E-05 |
