# Supplementary material for: A Comprehensive Analysis of miRNA/isomiR Expression with Gender Difference
Source: PLoS One. 2016 May 11;11(5):e0154955. doi: 10.1371/journal.pone.0154955 (PMC4864079; doi:10.1371/journal.pone.0154955)
Supplement: S3 Table — (DOCX) [file pone.0154955.s006.docx]

**Table S3.** **Statistical analysis results between paired tumor and normal samples, and male and females samples, using *t* test.**

| **miRNA** | **tissues** | **The first/second** | **The first/third** | **The first/fourth** |
| --- | --- | --- | --- | --- |
| miR-143 | **Female:** ucec-nt & ucec-tn | *t*=-0.4466, *P*=0.6596 | *t*=0.5144, *P*=0.6121 | *t*=-3.0487,*P*=0.0059 |
|  | **Male:** prad-nt & prad-tn | *t*=4.0472, *P*=0.0005 | *t*=6.1871, *P*<0.0001 | *t*=-2.9178, *P*=0.0080 |
|  | **Mix:** lusc-nt & lusc-tn | *t*=-1.7344, *P*=0.0968 | *t*=1.8381, *P*=0.0769 | *t*=-5.0451, *P*<0.0001 |
|  | **Mix:** thca-nt & thca-tn | *t*=-3.6783, *P*=0.0013 | *t*=-1.7857, *P*=0.0879 | *t*=-3.8685, *P*=0.0008 |
|  | **UCEC-nt & PRAD-nt:** Female & male | *t*=-0.7807, *P*=0.4433 | *t*=-2.9133, *P*=0.0081 | *t*=-2.9489, *P*=0.0074 |
|  | **UCEC-tn** &**PRAD-tn:** Female & male | *t*=2.8605, *P*=0.0091 | *t*=-0.5077, *P*=0.6167 | *t*=-0.6821, *P*=0.5023 |
|  | **LUSC-nt & THCA-nt** | *t*=2.3928, *P*=0.0257 | *t*=3.7356, *P*=0.0011 | *t*=0.8228, *P*=0.4195 |
|  | **LUSC-tn & THCA-tn** | *t*=1.0069, *P*=0.3249 | *t*=-0.5904, *P*=0.5610 | *t*=0.3549, *P*=0.7261 |
| miR-182 | **Female:** ucec-nt & ucec-tn | *t*=1.3947, *P*=0.1784 | *t*=1.2196, *P*=0.2368 | *t*=1.6695, *P*=0.1106 |
|  | **Male:** prad-nt & prad-tn | *t*=2.6727, *P*=0.0139 | *t*=-0.3501, *P*=0.7296 | *t*=-1.7780, *P*=0.0892 |
|  | **Mix:**lusc-nt & lusc-tn | *t*=5.8868, *P*<0.0001 | *t*=7.3121, *P*<0.0001 | *t*=5.0064, *P*=0.0001 |
|  | **Mix:**thca-nt & thca-tn | *t*=0.5194, *P*=0.6087 | *t*=2.6779, *P*=0.0137 | *t*=4.6570, *P*=0.0001 |
|  | **UCEC-nt & PRAD-nt:** Female & male | *t*=2.8809, *P*=0.0089 | *t*=4.1393, *P*=0.0005 | *t*=3.1326, *P*=0.0050 |
|  | **UCEC-tn** &**PRAD-tn:** Female & male | *t*=3.0655, *P*=0.0057 | *t*=0.6014, *P*=0.5537 | *t*=-0.1069, *P*=0.9159 |
|  | **LUSC-nt & THCA-nt** | *t*=-0.4405, *P*=0.6638 | *t*=-1.3538, *P*=0.1896 | *t*=-2.8430, *P*=0.0095 |
|  | **LUSC-tn & THCA-tn** | *t*=-5.5045, *P*<0.0001 | *t*=-4.8771, *P*=0.0001 | *t*=-4.0098, *P*=0.0006 |
